# Malaria mosquito control using edible fish in western Kenya: preliminary findings of a controlled study

**DOI:** 10.1186/1471-2458-7-199

**Published:** 2007-08-09

**Authors:** Annabel FV Howard, Guofa Zhou, Francois X Omlin

**Affiliations:** 1 Human Health Department, International Centre of Insect Physiology and Ecology (icipe), PO Box 30772-00100, Nairobi, Kenya; 2 Program in Public Health, College of Health Sciences, University of California, Irvine, CA 92697, USA

## Abstract

**Background:**

Biological control methods are once again being given much research focus for malaria vector control. This is largely due to the emerging threat of strong resistance to pesticides. Larvivorous fish have been used for over 100 years in mosquito control and many species have proved effective. In the western Kenyan highlands the larvivorous fish *Oreochromis niloticus *L. (Perciformes: Cichlidae) (formerly *Tilapia nilotica*) is commonly farmed and eaten but has not been previously tested in the field for malaria mosquito control.

**Methods:**

This fish was introduced into abandoned fishponds at an altitude of 1,880 m and the effect measured over six months on the numbers of mosquito immatures. For comparison an untreated control pond was used. During this time, all ponds were regularly cleared of emergent vegetation and fish re-stocking was not needed. Significant autocorrelation was removed from the time series data, and t-tests were used to investigate within a pond and within a mosquito type any differences before and after the introduction of *O. niloticus*. Mulla's formula was also used on the raw data to calculate the percentage reduction of the mosquito larvae.

**Results:**

After *O. niloticus *introduction, mosquito densities immediately dropped in the treated ponds but increased in the control pond. This increase was apparently due to climatic factors. Mulla's formula was applied which corrects for that natural tendency to increase. The results showed that after 15 weeks the fish caused a more than 94% reduction in both *Anopheles gambiae s.l*. and *Anopheles funestus *(Diptera: Culicidae) in the treated ponds, and more than 75% reduction in culicine mosquitoes. There was a highly significantly reduction in *A. gambiae s.l*. numbers when compared to pre-treatment levels.

**Conclusion:**

This study reports the first field trial data on *O. niloticus *for malaria mosquito control and shows that this species, already a popular food fish in western Kenya, is an apparently sustainable mosquito control tool which also offers a source of protein and income to people in rural areas. There should be no problem with acceptance of this malaria control method since the local communities already farm this fish species.

## Background

Mosquito control relies heavily on synthetic pyrethroids. Concern about the threat of strong forms of resistance [1] has stimulated renewed interest in alternative control methods including biological control and biopesticides. At present these methods are only operational against mosquito immatures [2-5], the best known being the use of *Bacillus thuringiensis *var. *israelensis *(*Bti*). *Bti *is effective against mosquito larvae [5] but cannot control the pupal stage, frequent repeat applications are needed [6] and it is expensive. Another biocontrol method, the use of larvivorous fish in appropriate water bodies, has been used in mosquito control for over 100 years [7] and can also be effective [2,8-10]. However, larvivorous fish offer advantages when compared to *Bti*. Fish feed on mosquito pupae and are generally self-sustaining, so in most cases do not require repeat applications. One disadvantage is that larvivorous fish can only be used under certain conditions conducive to their survival.

Almost 200 fish species are known to feed on mosquito larvae [11]. *Oreochromis niloticus *L. (Perciformes: Cichlidae) (formerly *Tilapia nilotica*) is a native African fish possessing mosquito control properties known since 1917 [12]. To our knowledge though, no field data has been published on its use for mosquito control. Under laboratory conditions this fish species has been shown to be larvivorous [13] with a 'marked interest in mosquito larvae' [8]. The fry actively pursue mosquito immatures [14] however when greater than 150 mm in length they prefer eating macrophytes [15]. Therefore, larger fish eat the plant material in which the mosquito immatures hide, allowing the fry to find them.

This fish species, commonly farmed by people in western Kenya as a source of protein and income, is a prolific breeder spawning every few weeks.

This study reports the first field trial data using *O. niloticus *for mosquito control. We found that abandoned (fish absent) fishponds had alarmingly high mosquito larval densities when compared to fishponds still containing fish (Howard et al, manuscript in review). We therefore investigated the long-term impact on mosquito densities of introducing *O. niloticus *into abandoned fishponds.

## Methods

### Study area

The study area was in Kisii Central District of western Kenya. The intervention site is 00°42 S, 34°46 E, at an elevation of 1,880 m above sea level with a population density of >1,000 people per km^2 ^[16].

Malaria in the area is endemic but highly seasonal with >2,000 paediatric cases annually in the district hospital [17]. The primary malaria vectors in the area are *Anopheles gambiae s.l*. and *Anopheles funestus *Giles.

**Figure 1:**
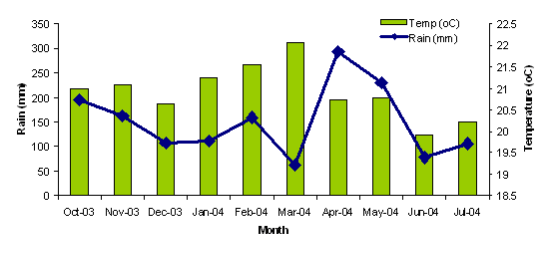
Mean monthly temperature and total monthly rainfall for the study area.

Rainfall averages over 1,500 mm annually with two wet seasons (February to June and September to November) and the mean annual maximum and minimum temperatures are 24°C and 14°C respectively [16]. Climatic data for the study period was obtained from the Kenya Agricultural Research Institute and is shown in Figure 1.

### Field intervention

The site has three abandoned fishponds within 150 m of each other. Pond A (104 m^2^) served as the untreated control and ponds C (128 m^2^) and D (72 m^2^) assigned for stocking with *O. niloticus*; each pond had a depth of 30 cm. These fishponds had been re-constructed under the instruction of a Fisheries Department (FD) officer.

Entomological assessments were carried out by taking five larval dips (2.5 litres total volume) randomly from the edges of each pond, with at least one dip from each side. These assessments were carried out 5–7 days a week and began on the 1^st ^October 2003. Anophelines and culicines were distinguished, with anophelines identified to species level using a morphological key [18].

On the 14^th ^January 2004 one to two month old *O. niloticus *from the local FD hatchery in Kisii town were stocked in ponds C and D at a rate of two per m^2 ^pond surface area. FD representatives instructed the fishpond owners on fish husbandry and pond maintenance.

The three ponds were cleared of vegetation on a weekly basis and treated identically during the nine month study period. The fish were neither harvested nor the ponds restocked.

### Statistical analysis

Analysis was carried out on the data 15 weeks before and 15 weeks after fish introduction into ponds C and D. We used the one-lag autoregression model to determine the autocorrelation of the time series data. Significant autocorrelation was removed along with the deterministic drift term. We then used paired t-tests to see if the two treated ponds were significantly different before fish introduction. If there was no difference the data from the ponds were pooled. We also used t-tests to investigate within the pond and within the mosquito type any differences before and after fish introduction. All tests were carried out at the 5% significance level.

Using the raw data, the percentage reduction of mosquito immatures in ponds C and D after fish introduction was calculated using Mulla's formula [19]. This formula corrects for any changes seen in the control pond that would presumably also have occurred in the treated ponds in the absence of the intervention.

## Results

**Figure 2:**
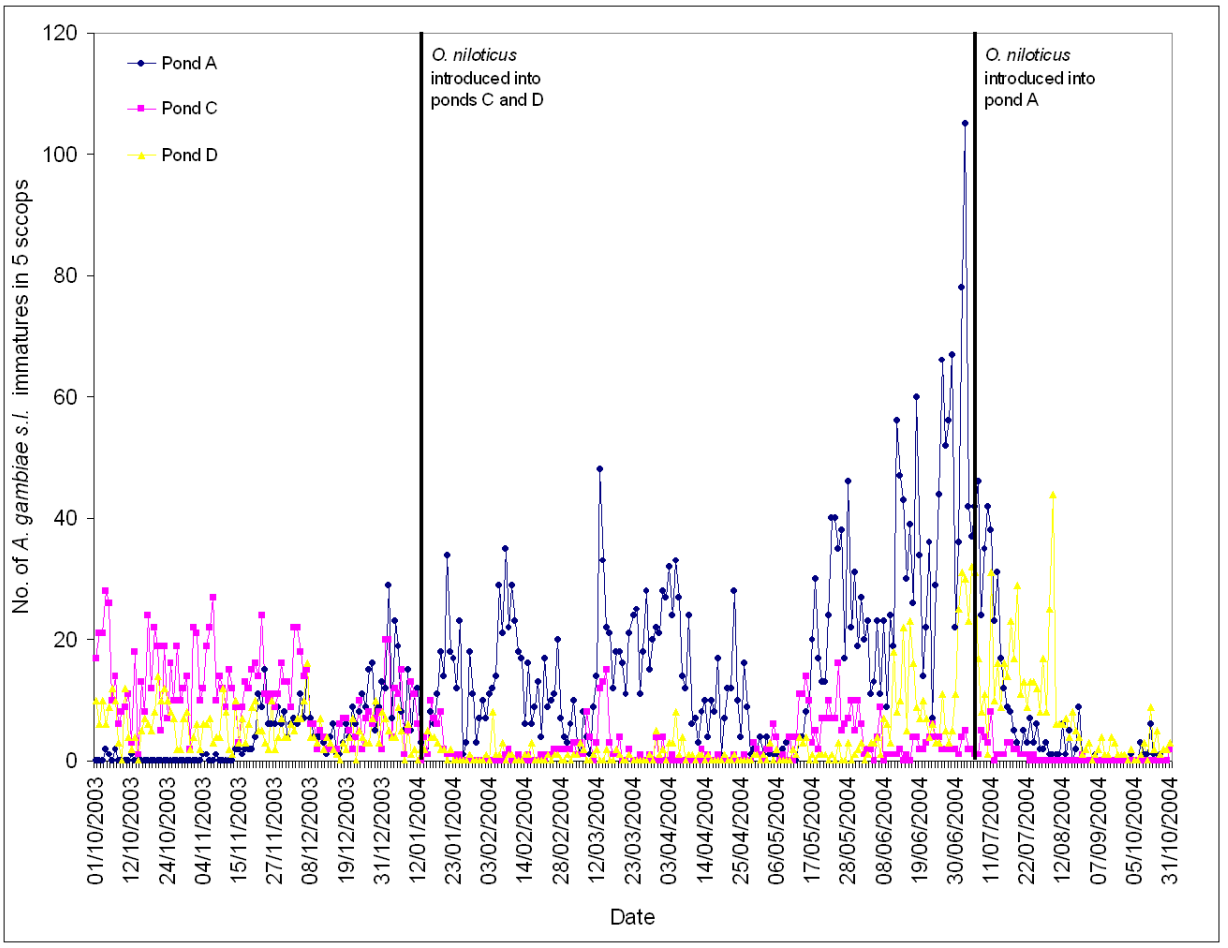
*A. gambiae s.l*. numbers in the control and treated ponds before and after fish introduction.

The *A. gambiae s.l*. numbers in ponds A, C and D for 15 weeks prior to and 41 weeks after *O. niloticus *introduction into ponds C and D are presented in Figure 2. Ten days after fish introduction, no mosquitoes were found in pond C and a clear difference can be seen between ponds A and C for the next six months. Pond D shows a similar pattern.

The mean immature mosquito densities before autocorrelation removal are presented in Table 1, along with the Mulla's formula [19] results. It can be seen that after fish introduction, the numbers of all mosquito types increased in the control pond, and decreased in the treated ponds. High percentage reductions were found for *A. gambiae s.l*. and *A. funestus*. For culicines it was lower but the reduction was still >75%.

**Table 1:** Immature mosquito densities before and after *O. niloticus *introduction into ponds C and D

	Pond A^*a*^		Pond C			Pond D		
Mosquito species	Before^*b*^	After^*c*^	Before	After	% Reduction^*d*^	Before	After	% Reduction

*A. gambiae s.l*.	4.50 ± 0.56	14.08 ± 0.93	11.38 ± 0.67	1.53 ± 0.28	95.8	5.40 ± 0.34	1.00 ± 0.15	94.1
*A. funestus*	0.06 ± 0.04	2.45 ± 0.47	0.57 ± 0.1	0.43 ± 0.14	98.3	0.61 ± 0.14	0.56 ± 0.10	97.5
Culicines	2.34 ± 0.38	4.51 ± 0.44	2.81 ± 0.47	0.72 ± 0.18	86.7	1.29 ± 0.21	0.63 ± 0.13	75.4

^*a *^Pond A was the control pond, ponds C and D were treated

^*b *^'Before' refers to the 15 weeks before fish introduction into ponds C and D

^*c *^'After' refers to the 15 weeks after fish introduction into ponds C and D

^*d *^Percentage reduction was corrected for observed natural increases in the control pond using Mulla's formula [19].

Significant autocorrelation was detected in all ponds for all mosquito species except for culicines in pond C. However, the first two data points for culicines in pond C were removed from the analysis in order to use the same number of data points as ponds A and D. No significant differences between ponds C and D for *A. gambiae s.l*. or *A. funestus *before fish introduction were found so the data were pooled.

After autocorrelation removal, when comparing within a pond the pre- and post-intervention data, fish introduction caused highly significant reductions of *A. gambiae s.l*. in the treated ponds (t_127 _= 3.81, p < 0.0002) and culicines in pond C (t_128 _= 4.16, p < 0.0001), and a significant reduction of culicines in pond D (t_162 _= 1.97, p < 0.05).*A. funestus *numbers in the treated ponds decreased but not significantly (t_129 _= 1.13, p = 0.26). In the control pond the mosquito numbers increased for all species but not significantly so for *A. funestus *(t_104 _= 1.79, p = 0.08).

In view of the high *A. gambiae s.l*. densities in pond A we introduced *O. niloticus *into this pond once the experiment was complete. These densities dropped from 105 mosquitoes in five dips just before fish introduction, to one mosquito in five dips two weeks later and remained low for the next three months. However, without a contemporary untreated control we cannot be sure this was solely because of the fish.

## Discussion

Our field data demonstrates, for the first time, that the introduction of *O. niloticus *into fishponds immediately and significantly reduces the numbers of *A. gambiae s.l*. and culicine larvae in treated ponds. Fifteen weeks after fish introduction, the impact on both anopheline species was a >94% reduction after correction for the natural increase expected. By contrast, Mohamed [9] reported that *Oreochromis spilurus spilurus *introduced into water storage containers in Somalia showed a mean reduction of 52.8%, while *Gambusia affinis *produced a 87.8% decline in mosquito larvae in rice fields [10]. However these results represent both different fish species and ecological settings.

The large percentage reductions in the treated ponds, as calculated with the raw data using Mulla's formula, was a combined effect of the decrease of mosquito numbers in the treated ponds and the increase in the control pond.

Fifteen weeks after fish introduction into ponds C and D there was an increase of all mosquito species in the control pond. This was most likely due to low rainfall leading to a reduction in the number of alternative oviposition sites. When the rainfall increased in April, the number of mosquitoes in the control pond decreased dramatically. This negative correlation of mosquito larval densities with rainfall has been previously found in Kenyan rice fields [8].

The decrease of the mosquito numbers in the treated ponds might be directly (by predation) and/or indirectly (by oviposition avoidance by mosquitoes) due to the fish. Evidence that the fish were directly responsible comes from observed minor peaks in the mosquito densities in ponds C (from 13^th ^May) and D (from 8^th ^June) that corresponded with the time when the fish were mature enough to start reproduction. When reproducing, neither male nor female *O. niloticus *feed [14], which would explain the temporary peaks, contrary to the overall downward trend.

A tendency of ovipositing mosquitoes to avoid ponds containing fish has previously been found with *Anopheles punctipennis *[20], and culicine mosquitoes [21,22]. However, in a separate study of 261 fishponds we found no significant difference between the number of fish-present and fish-absent fishponds containing mosquito immatures (Howard et al, manuscript in review). This suggests that mosquitoes do not avoid fish-containing water in this area.

Given the already proven larvivorous behaviour of *O. niloticus*, the peaks of mosquitoes during fish reproduction, the findings in the separate study, and also taking into account the climatic relationship of the mosquito increase in the control pond, it seems likely that the fish are directly controlling the mosquito numbers in the treated ponds through predation.

*A. funestus *was not significantly decreased after fish introduction. The large percentage decrease calculated is a result of the 40-fold increase in the control pond, indicating a strong tendency for natural increase in the local population. The fish apparently ate enough *A. funestus *larvae to counterbalance this natural increase, but not enough to produce an overall reduction.

The fact that we still recorded larvae in the treated ponds does not mean these ponds were still producing adult mosquitoes. From other sites in Kisii Central District, we have noticed disproportionate numbers of first and second instar mosquito larvae in fishponds containing fish, indicating that fish are more likely to eat the older, larger instars. This was also found with other fish species in Somalia where after fish introduction only first and second instar larvae were present [9], and in laboratory and field studies in China [23].

In the year 2000, Kisii Central District was reported to have 554 fishponds in an area of 649 km^2 ^[16] while the neighbouring district of Nyamira was reported to have 1,046 fishponds in 896 km^2 ^[24]. It is likely that these are under-representations of the actual fishpond numbers in the two rural districts as the topography is hilly with poor road networks, which make locating fishponds difficult. Given the large size of the fishponds and the fact that they contain water all year round, they could be considered a fairly significant producer of malaria vectors in this area of western Kenya. Unfortunately our results do not show the proportion of the adult mosquito population that is produced by the fishponds, relative to the more classic *An. gambiae s.l*. immature habitats such as small transient pools of water [25] which are unsuitable for *O. niloticus*. As such we are unable to say how effective this control method would be in reducing the adult mosquito population in a given area. However, our results show that *O. niloticus *fish were so effective in reducing immature mosquito populations in the fishponds studied, that there is likely to be a noticeable effect on the adult mosquito population in the area. This will be addressed in future studies.

Benefits of larvivorous fish are that the mosquito larvae cannot build up a physiological resistance, also, fish populations are generally self-sustaining and do not depend on the presence of larvae. By contrast survival of other biological control agents is often dependant on the mosquito population not being entirely eliminated [26]. In addition some *Anopheles *larvae have significantly prolonged developmental times in the presence of fish and emerge as smaller adults [27]. Smaller females, in turn, have significantly reduced host seeking [28] and produce smaller egg batches [29], making them less efficient malaria vectors.

As well as protection from mosquito-borne diseases such as malaria, *O. niloticus *has additional benefits. The fish are relatively inexpensive and six months after stocking the larger fish can be harvested, providing a sustainable source of income and protein to rural farmers. This fish is already farmed and eaten in this region of Kenya so acceptance by both the local communities and the administrative sectors should pose no problem.

Larval control has long been neglected. However, it can be an effective control tool due to the low mobility of larval mosquitoes [30], especially where the principle breeding habitats are man-made [31-33] and can be easily identified [34].

We are undertaking larger scale field trials in different ecological settings, whilst monitoring mosquito predator numbers and diversity to investigate any detrimental impact by the fish. In villages both with and without fish introductions, it in necessary to monitor adult mosquito densities and malaria incidence to confirm its use as a malaria control tool.

## Conclusion

In conclusion, our results indicate that *O. niloticus *can dramatically reduce mosquito larval densities in fishponds for at least six months and that this reduction is directly through predation. The relative population density of *A. gambiae s.l*., a very efficient malaria vector, was reduced by 94% and this reduction was statistically highly significant.

## Competing interests

The author(s) declare that they have no competing interests.

## Authors' contributions

AFVH analysed part of the data and wrote the manuscript. GZ analysed part of the data. FXO designed the study and helped to draft the manuscript. All authors read and approved the final version of the manuscript.

## Pre-publication history

The pre-publication history for this paper can be accessed here:


